# Supplementary material for: Novel Clustering Methods Identified Three Caries Status-Related Clusters Based on Oral Microbiome in Thai Mother–Child Dyads
Source: Genes (Basel). 2023 Mar 3;14(3):641. doi: 10.3390/genes14030641 (PMC10048127; doi:10.3390/genes14030641)
Supplement: Supplementary file 1 [file genes-14-00641-s001.zip › genes-2223500-supplementary.pdf]

Supplementary Materials

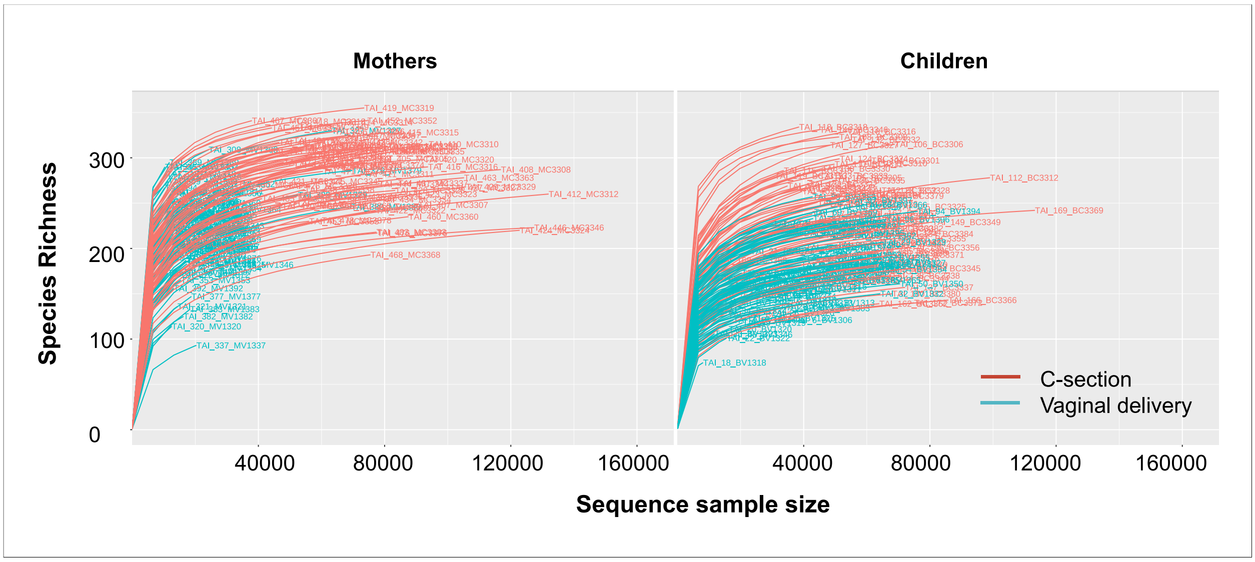

Figure S1. Rarefaction curve of mother and children's salivary oral microbiome.
